# Effects of Volatile Flavour Compound Variations on the Varying Aroma of Mangoes ‘*Tainong*’ and ‘*Hongyu*’ during Storage

**DOI:** 10.3390/molecules28093693

**Published:** 2023-04-25

**Authors:** Huiwen Xie, Lanhuan Meng, Ying Guo, Hongmei Xiao, Libo Jiang, Zhengke Zhang, Haichao Song, Xuequn Shi

**Affiliations:** 1 College of Food Science and Engineering, Hainan University, Haikou 570228, China; 2 Department Food Science and Human Nutrition, Institute for Integrative Toxicology, Michigan State University, East Lansing, MI 48824, USA; 3 Sanya Institute of Nanjing Agricultural University, Sanya 572024, China; 4 College of Food Science and Technology, Nanjing Agricultural University, Nanjing 210095, China; 5 School of Life Sciences and Medicine, Shandong University of Technology, Zibo 255000, China; 6 School of Life Sciences, Hainan University, Haikou 570228, China

**Keywords:** characteristic aroma substances, intact mango, cut mango, GC-IMS, HS-SPME-GC-MS, storage, marketing time

## Abstract

The aroma, taste, and flavour profiles of mango cultivars vary, directly influencing their marketability and consumer acceptance. In this study, we explored the effects of volatile organic compounds (VOCs) on the distinct aromas of two mango cultivars during storage using GC-IMS and HS-SPME-GC-MS combined with OPLS-DA analysis. Our findings revealed that the terpene and aldehyde contents were higher in the ‘*Tainong*’ mango cultivar, compared to the ‘*Hongyu*’ mango, while the ester content was lower. The aroma was attributed to the presence of terpinolene, 2-nonenal, delta-carene, and alpha-phellandrene in the early stages of storage, and later—between 5 and 11 days—to ethyl acetate, ethyl butyrate, and ethyl propanoate. Further analysis of characteristic VOCs using OPLS-DA demonstrated and explained the strong grassy aroma of the ‘*Tainong*’ mango, and the strong fruity and sweet aromas of the ‘*Hongyu*’ mango. Additionally, esters mainly accumulated during the later periods of storage, especially propyl butyrate, which was produced and accumulated when fruit quality deteriorated in the later storage period. Our study provides a theoretical basis for detecting mango VOCs during storage to determine the appropriate marketing time for the two mango cultivars and enables informed consumer choice.

## 1. Introduction

Mango (*Mangifera indica* L.) is one of the most consumed and commercially significant tropical fruits, with more than 26 million tonnes produced annually worldwide [1]. ‘*Tainong*’ and ‘*Hongyu*’ are the main commercial mango cultivars produced in Changjiang County, Hainan Province, China. Mangoes are suitable for long cultivation in slightly acidic soils, or sandy loamy soils; they are not cold-tolerant and need to be cultivated at temperatures above 15 °C [2]. Mangoes are prone to browning of the flesh, degradation of texture, or changes in flavour during post-harvest ripening, shipping, and storage, resulting in the disposal of large quantities of substandard fruit each year [2,3,4].

The aroma of a fruit is an important indicator of fruit quality because the aroma components might determine consumer acceptance and marketability of mangoes. Many factors can affect mango flavour and there are significant differences between different origin and varieties [5]. Mangoes that are not ripe at the time of picking may provide a longer shelf life [6,7], but mangoes are not ripe enough to satisfy consumers’ preference for firmness, and do not provide acceptable volatiles and flavour [8,9]. Moreover, dense fruit arrangement can induce the degradation of terpenoids, reduce the formation of lipoxygenase pathway reaction products, and reduce the aroma and flavour of mango [4]. In the past, the volatiles of different mango cultivars and hundreds of other compounds have been identified, including aldehydes, alcohols, esters, ketones, and terpenes [5,10,11]. The limonene, beta-ocimene, beta-caryophyllene, and alpha-terpinene released by mango during ripening are important in some varieties, and they all contribute to the characteristic mango flavour [6]. The content of sesquiterpene varies greatly among different varieties [6,12]. While furanol, *(Z)*-2, 6-dimethyl-3, 5, 7-octtriene-2-alcohols, and all lactones are qualitatively important [13]. Studies have shown that alpha-terpinene, nonanal, limonene, and 2-methylbutyraldehyde could affect the negative pleasure perception of mango [14,15]. Some fruity esters, including ethyl butyrate, ethyl 3-methylbutyrate, ethyl 2-methylpropionate, and ethyl 2-methylbutyrate, are generally effective aromatically active compounds with a fruity character in mangoes [5].

During the ‘ripening period’, the colour, firmness, size, shape, and aroma of the fruits change significantly. Similarly, the flavour, taste, appearance, and nutritional components of mangoes alter during storage based on the variety and characteristics of the commodity. Each volatile organic compound (VOC) has a different odour. Their combinations, concentrations, and ratios give the fruit unique aroma characteristics through cumulative, synergistic, and masking effects. Terpenes are the most abundant aromatic substance in Kent mangoes, followed by esters and other compounds after 48 h of ripening [4], but the dynamic changes in VOCs should be studied in more cultivars.

Gas chromatography-ion mobility spectrometry (GC-IMS) has been developed in recent years to simultaneously obtain the composition of flavour-related compounds and the quality of samples [16,17]. The combination of HS-GC-IMS and HS-SPME-GC-MS analyses is an intuitive method for sample differentiation based on VOCs and could comprehensively profile VOCs in mango samples.

In this study, we measured the skin colour, fruit hardness, soluble solid concentration, titratable acidity, and ethylene production. Additionally, we calculated the respiration rate and identified the VOCs during the storage of two mango cultivars to further understand mango aroma and quality changes during ripening. Furthermore, we studied differences in characteristic VOCs between intact and cut mango by GC-IMS and OPLS-DA analysis; this helped assess mango fruit quality changes during storage by detecting the changes in the VOC content and composition. This study aims to provide a theoretical basis for the storage management of mangoes and to determine the appropriate time to market the mango fruits of the two cultivars.

## 2. Results and Discussion

### 2.1. Physicochemical Analysis

**Figure 1:**
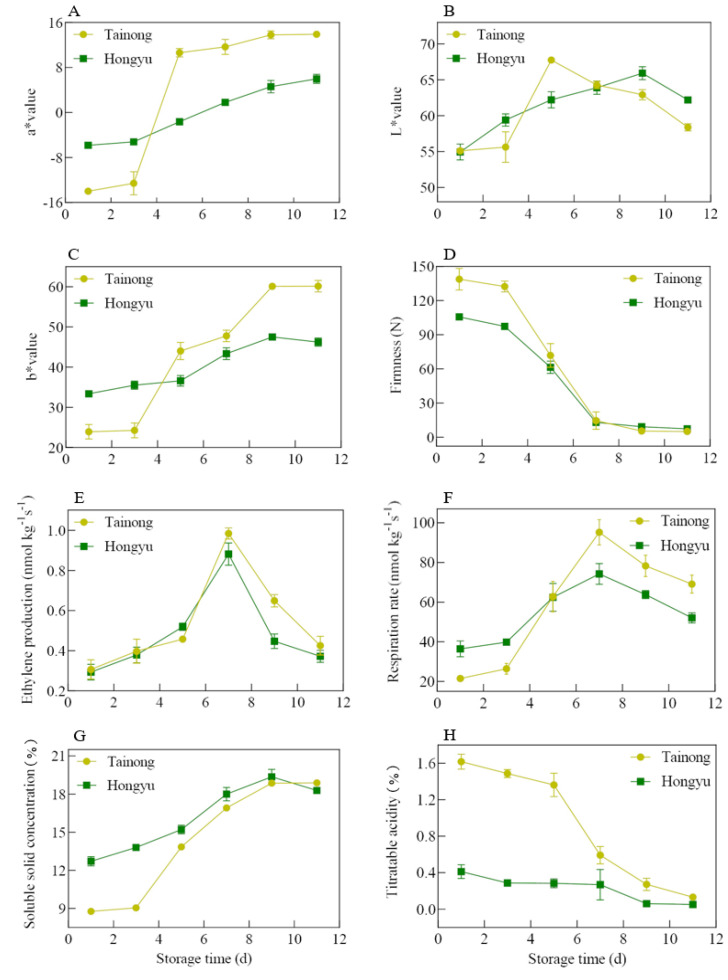
Difference of a* value (**A**), L* value (**B**), b* value (**C**), firmness (**D**), ethylene production (**E**), respiration rate (**F**), soluble solid concentration (**G**), and titratable acidity (**H**) between ‘*Tainong*’ and ‘*Hongyu*’. Error bars represent the standard deviation of means.

**Figure 2:**
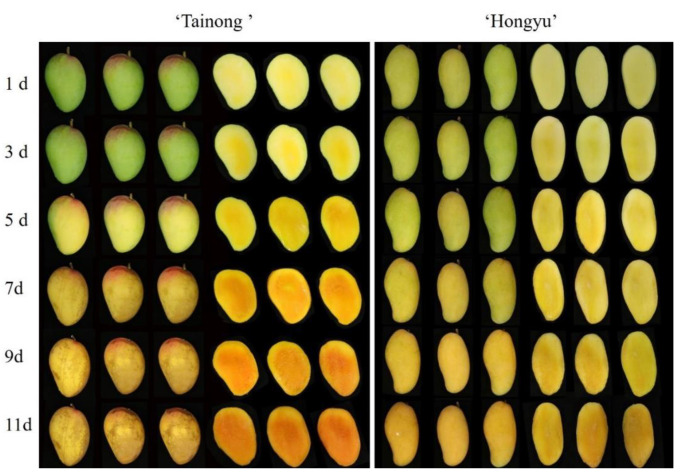
The appearance of mango (‘*Tainong*’ and ‘*Hongyu*’) after storage of different periods.

The characteristics of aroma, taste, and flavour differ depending on the maturity, size, colour, and firmness of fruit, which affect post-harvest fruit quality and consumer acceptance [1,18,19]. The respiration rate of fruit, including the oxidation of carbohydrates, organic acids, and other organic molecules in the cells and the concurrent energy production, is closely related to the ripening and senescence of fruits [18]. Moreover, ethylene released during storage results in the softening of the fruit and is associated with an increased incidence of black spots in mangoes [1]. As shown in Figure 1A–C, both indices increased in ‘*Tainong*’ and ‘*Hongyu*’ with storage time. The a* and b* values of ‘*Hongyu*’ mango increased at a slower rate compared with those for the ‘*Tainong*’ mango. The L* value of ‘*Tainong*’ mango showed a sharp change after three days and then fall rapidly after five days. The ‘*Tainong*’ mango had a darker colour and a large area of black spots after storage for 7 d, while the ‘*Hongyu*’ mango still possessed market value after 11 d (Figure 2). The dark spots of ‘*Tainong*’ mango might be due to the rapid fading of mango lustre due to rapid maturation, wilting, and excessive growth of fungal mycelia in mango [1]. The ethylene production and respiration rate of mangoes in ‘*Tainong*’ were higher than those in ‘*Hongyu*’ from 1–5 d, with the former reaching peak respiration rate after 7 d (Figure 1E,F).

A mango firmness value of 5 N is usually regarded as the threshold below which the fruit is inedible [18]. After 11 d, this value of ‘*Tainong*’ and ‘*Hongyu*’ were 4.98 N and 7.39 N, making them nonedible and edible, respectively. This value of ‘*Tainong*’ was higher than ‘*Hongyu*’ for the first three days of storage but decreased rapidly after three days, reaching the threshold at day seven of storage (Figure 1D). Similar results have been previously reported for other mangoes [20,21,22]. This rapid decrease in firmness may be due to the degradation of the cell wall [17]. These results indicate that during storage, the cell walls soften and the intracellular enzymes react with other substrates to form VOCs [23].

Titratable acidity and soluble solid concentration (mostly consisting of carbohydrates, acids, and a few trace quantities of dissolved substances) are crucial quality indicators for the flavour of fresh mango products and the flavour development during ripening [1,20]. In this present study, the titratable acidity of ‘*Tainong*’ decreased to about 1/3 of the initial value after 5 d, while the titratable acidity of ‘*Hongyu*’ remained stable (Figure 1G,H) [1]. The high respiration rate of the fruit was proportional to the large consumption of organic acids expected to reduce titratable acidity. The decrease in titratable acidity during storage was attributed to the fruit’s use of acids as respiratory substrates, converting them to sugars through gluconeogenesis [21,24]. Furthermore, we observed a slight increase in soluble solid concentration during the ripening of the mangoes. The ‘*Tainong*’ fruits displayed stronger colours with respect to ‘*Hongyu*’ fruits in both skin and flesh. Therefore, the acidic taste was more intense in ‘*Tainong*’ compared with ‘*Hongyu*’ mangoes. In contrast, ‘*Hongyu*’ fruit had a sweeter taste. Mangoes are typically of better quality in Western nations, with lower fibre content, intense sweetness, balanced acidity, high fluid, and low terpene flavour [5]. According to our results and market feedback [21], the best fruit quality of ‘*Tainong*’ and ‘*Hongyu*’ mangoes was seen at 5–7 and 7–9 days post-harvest, respectively.

### 2.2. Assessing VOCs in Cut Mangoes Using GC-IMS

**Figure 3:**
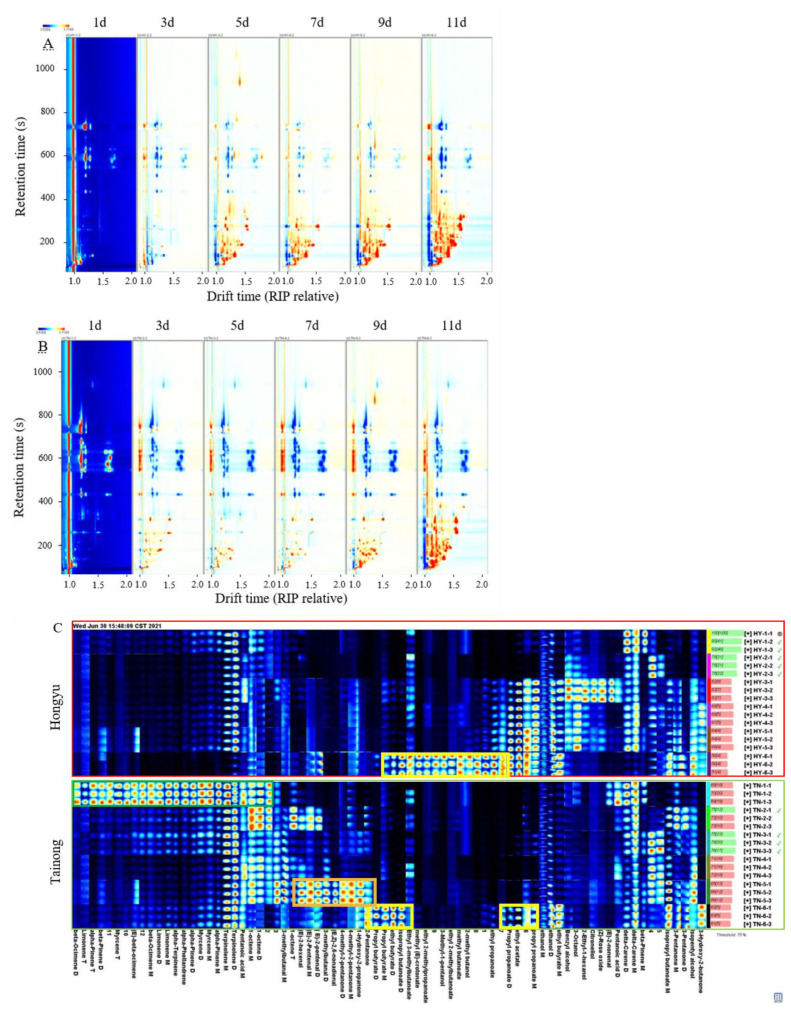
The topographic plots of GC-IMS spectra of ‘*Hongyu*’ cut mango (**A**), and ‘*Tainong*’ cut mango (**B**), with different storage time. The gallery plot (**C**) of VOCs in cut mangoes with different storage time. (The same row represents the signal peaks of different VOCs detected by a sample and the same column represents the signal peaks of a VOC.) HY-1-1, HY-1-2, HY-1-3: ‘*Hongyu*’, on the 1st d; HY-2-1, HY-2-2, HY-2-3: ‘*Hongyu*’, on the 3rd d; HY-3-1, HY-3-2, HY-3-3: ‘Hongyu’, on the 5th d; HY-4-1, HY-4-2, HY-4-3: ‘*Hongyu*’, on the 7th d; HY-5-1, HY-5-2, HY-5-3: ‘Hongyu’, on the 9th d; HY-6-1, HY-6-2, HY-6-3: ‘*Hongyu*’, on the 11th d; TN-1-1, TN-1-2, TN-1-3: ‘*Tainong*’, on the 1st d; TN-2-1, TN-2-2, TN-2-3: ‘*Tainong*’, on the 3rd d; TN-3-1, TN-3-2, TN-3-3: ‘*Tainong*’, on the 5th d; TN-4-1, TN-4-2, TN-4-3: ‘*Tainong*’, on the 7th d; TN-5-1, TN-5-2, TN-5-3: ‘*Tainong*’, on the 9th d; TN-6-1, TN-6-2, TN-6-3: ‘*Tainong*’, on the 11th d.

The formation of VOCs during mango ripening enables volatile and aromatic compounds to evoke human senses, including taste [4]. In this study, the GC-IMS method was used to identify the VOCs and flavour substances in mangoes and their variations during storage [25]. The variation in VOCs during storage at six different stages is presented in Figure 3. The differential graphs (Figure 3A,B) were compared for the changes in VOCs during storage using a 1 d sample as the baseline. The bulk of the red signals from ‘*Tainong*’ and ‘*Hongyu*’ cut mangoes during early storage emerged in the 50–200 s retention time and drifted from 1.0 to 1.5, while the retention and drift times of ‘*Tainong*’ cut mango under post-storage increased rapidly at 400 s and drifted to 1.7, respectively. This result indicates that many VOCs were produced during storage. The increase in VOCs is believed to be due to the dynamic and continuous synthesis of volatile compounds [26]. With increasing storage period, the blue signals in the 400–800 s retention time range also increased.

Although the topographic map shows a trend towards VOCs, it is difficult to accurately quantify the substances on the map. Fingerprints have been used to solve this problem [26]. To intuitively compare the changes in VOCs in the storage of the two mango varieties, fingerprints of the two varieties were established (Figure 3C) using GC-IMS to detect 182 signal peaks, with 72 signal peaks in cut fruit and 110 signal peaks in intact fruit. Among them, 65 and 86 signal peaks were identified and marked with known substances, respectively (Table 1). In particular, 32 VOCs were detected in ‘*Tainong*’ and ‘*Hongyu*’ cut mangoes. There were ten terpenes in ‘*Tainong*’, which made them the most prominent class of compounds. The most abundant compound was delta-carene, followed by terpinolene, limonene, myrcene, and alpha-pinene. The most abundant compounds were esters. A total of 11 esters were found in ‘*Hongyu*’, with ethyl acetate being the most abundant, followed by propyl propanoate, ethyl propanoate, and isopropyl butanoate. Reportedly [11], esters, terpenes, and aldehydes are the main VOCs during mango storage. Most terpenes have a natural aroma, similar to the characteristic aroma of lemons [23]. Among alpha-pinene, beta-pinene, limonene, beta-ocimene, alpha-terpinene, alpha-phellandrene, *(E)*-beta-ocimene, and myrcene—content in the ‘*Tainong*’ cut mango was higher than in the ‘*Hongyu*’ cut mango. In addition, ‘*Tainong*’ possessed the highest terpene content after 1 d of storage (green frame), whereas the content of terpenes in the subsequent detection process was lower than that on day one. This may be because the aroma measured before ripening mainly comprises terpenes [27].

Similarly, the contents of alpha-pinene, beta-pinene, myrcene, limonene, terpinolene, delta-carene, alpha-terpinene, alpha-phellandrene, and other substances were high in ‘*Hongyu*’ cut fruit at the initial stage of storage. Most were almost undetectable after 11 days. In contrast, beta-ocimene and *(E)*-beta-ocimene were detected only in ‘*Tainong*’. Thiruchelvam [28] demonstrated that *(E)*-beta-ocimene is the main volatile compound in mangoes. Most terpenes have the same aroma as flowers and plants [28]. Generally, the contents of terpenes detected in ‘*Hongyu*’ cut fruit were relatively low during storage.

The ester content gradually increased during storage and reached its maximum at 11 days (yellow frame). These esters are usually formed by the beta-oxidation pathway during ripening [4]. Although the ester composition in the two mango varieties was different, the main active aromatic esters were similar. For example, ethyl acetate, ethyl butyrate, ethyl propanoate, isopropyl butanoate, methyl crotonate, propyl butyrate, and propanoate were detected in both species. Some esters were only detected in ‘*Hongyu*’ cut mango, for example, ethyl 2-methylbutanoate, ethyl 3-methylbutanoate, and methyl butanoate. Among them, the contents of ethyl acetate, ethyl propanoate, and propyl propanoate in ‘*Hongyu*’ cut mango were higher than those in the ‘*Tainong*’ cut fruit after 5 d. It has been reported that ethyl butyrate and ethyl acetate are responsible for the fruity flavour of mangoes and are the major volatile products of ripe fruit [29,30]. Some studies showed that ethyl 2-methylbutanoate and ethyl 3-methylbutanoate played an important role in the overall “fruity taste” of mango, affecting the aroma compounds of mango fruit flavour [31,32]. This might be the reason for the superior fruit flavour of the ‘*Hongyu*’ cut mango compared with the ‘*Tainong*’. Aldehydes significantly increased in the ‘*Tainong*’ cut fruit after 9 d (orange frame); this might occur due to aldehydes produced by fatty acid oxidation or amino acid metabolism during fruit storage [23,31].

Note that propyl butyrate, ethyl butyrate, isopropyl butanoate, methyl crotonate, ethyl 3-methylbutanoate, ethyl 2-methylpropanoate, ethyl 2-methylbutanoate, methyl butanoate, ethyl propanoate, propyl propanoate, and ethyl acetate were rapidly produced by both ‘*Hongyu*’ and ‘*Tainong*’ cut mangoes when their edible value was above the threshold (11 d) (Table 1), while *(E)*- 2-hexenal, *(E)*- 2-pentenal, 3-methylbutanal, *(E,Z)*- 2,6-nonadienal, 4-methyl-2-pentanone, 1-hydroxy-2-propanone, and 2-pentanone were rapidly produced by ‘*Tainong*’ when its edible value was above the threshold (9 d). The increase in these VOCs during storage indicated the inferior quality of the mangoes.

### 2.3. Volatile Compounds in Intact Mango by GC-IMS

**Figure 4:**
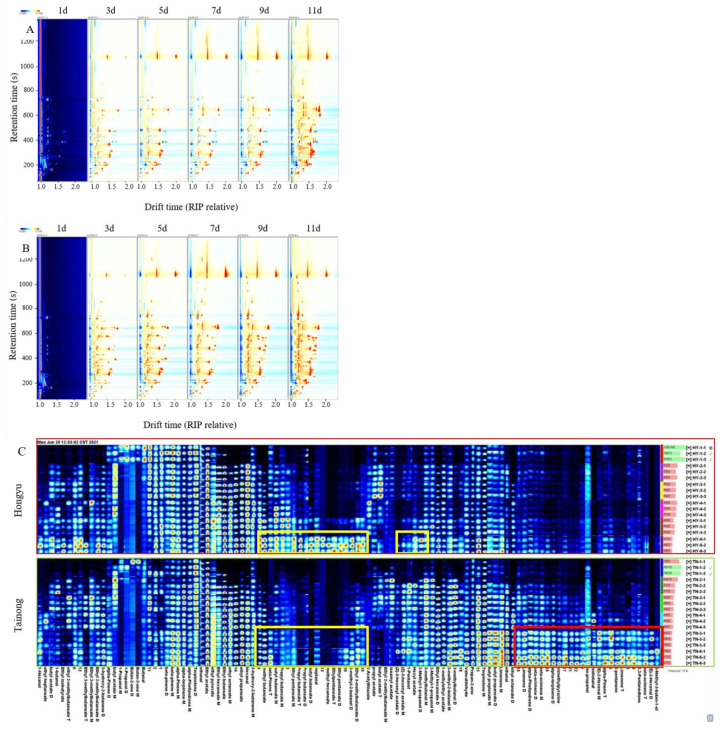
The topographic plots of GC–IMS spectra of ‘*Hongyu*’ intact mango (**A**), and ‘*Tainong*’ intact mango (**B**), with different storage time. The gallery plot (**C**) of VOCs in intact mangoes with different storage time.

In ‘*Tainong*’ intact mango, most of the red signals were between 50 and 800 s in retention time and between 1.0 and 1.8 s in drift time (Figure 4A,B). Remarkably, the ‘*Tainong*’ intact mangoes increased gradually during storage. Using the fingerprint of the two varieties (Figure 4C), 51 VOCs were detected in ‘*Tainong*’ intact fruit, and 48 VOCs were detected in ‘*Hongyu*’ intact fruit. The types of VOCs in intact mangoes were higher than those in cut mangoes, but the mangoes had fewer terpene VOCs (seven). The most prominent compound class was esters, with a total of 21 and 23 in ‘*Tainong*’ and ‘*Hongyu*’, respectively. The aldehyde and terpene contents in the ‘*Tainong*’ intact fruit were higher than those in the ‘*Hongyu*’ intact fruit. Unlike in the cut fruit, terpenes detected in intact fruits were found in both varieties. Interestingly, the terpene compounds in cut mango fruit only appeared in the early stage of storage, while the terpene VOCs in the intact fruit accumulated gradually during storage. It has been reported that terpenes are the main aroma components of mango, and these substances increase gradually with the extension of detection time [4]. Aldehydes are the main components responsible for the grassy and green flavours [10]. At the beginning of storage, the detected intensity of aldehydes was low and began to increase from day three. During the intact fruit storage, the detected signal intensity of aldehydes in the ‘*Tainong*’ was higher than that in the ‘*Hongyu*’. Notably, *(E)*-2-hexenal was only detected in the ‘*Tainong*’ intact fruit, which had a very pleasant, more spice-like green aroma, with fatty-grassy, and green-fruity notes [29], which might be related to the production of waxy oil substances on the surface of ‘*Tainong*’ fruit at later stages of storage. In contrast, the terpentine and green flavours of mangoes resulted in a relatively low enjoyment preference associated with aldehydes and terpene content. The contents and types of terpenes in the ‘*Tainong*’ cut fruit were the highest. This makes the ‘*Tainong*’ aroma worse in the early stage of storage. Moreover, ethyl pentanoate and methyl hexanoate were not detected at the initial stage of the ‘*Tainong*’ intact fruit storage, and ethyl pentanoate was detected as the fruits approached decay. With the extension of storage time, many esters accumulated in the ‘*Hongyu*’ intact fruit, exceeding their corresponding contents in the ‘*Tainong*’ fruit.

As the VOCs increased, compounds such as 3-methyl-3-buten-1-ol, *(E)*-2-hexenal, beta-ocimene, 2,3-pentanedione, limonene, 2-pentanone, alpha-pinene, isobutanal, iso-propanol, trimethylpyrazine, alpha-terpinene, and alpha-phellandrene were rapidly produced in the ‘*Tainong*’ intact mango after day seven. Meanwhile, ethyl 3-methylbutanoate, 3-methyl-2-butanol, ethyl propanoate, methyl hexanoate, heptanal, butyl butanoate, propyl butyrate, beta-pinene, and methyl butanoate were drastically produced for the ‘*Hongyu*’ intact mango in storage after 11 d (Table 1). The results indicated that the generation of these VOCs was related to the inferior quality of the mangoes.

### 2.4. Assessing the VOCs in Mango Using HS-SPME-GC-MS

A total of 92 VOCs were identified using HS-SPME-GC-MS to comprehensively understand the variation in VOCs. They included alcohols (22), esters (13), aldehydes (18), ketones (10), and terpenes (29). The combination of HS-SPME-GC-MS and HS-GC-IMS enhanced the VOC recognition capabilities [33]. In this present study, HS-SPME-GC-MS was found to be more sensitive to terpenes and aldehydes, whereas HS-GC-IMS measured esters more sensitively. As shown in Table 1, for the VOCs identified in mangoes, terpenes were predominant in terms of content and variety. After storage for 3 d, a large number of esters were identified in the ‘*Tainong*’, while the esters in the ‘*Hongyu*’ accumulated more gradually during storage. Meanwhile, the most abundant aldehydes and ketones were the C6 aldehydes 3-hexenal, 2-methylpent-4-enal, 2-hexenal, and nonanal. Among them, the C6 and C10 compounds, 3-hexen-1-ol, nerol, and 1-hexanol, were found to have the highest concentrations. Notably, pentyl acetate, methyl acetate, butyl hexanoate, methyl butyrate, propyl butyrate, isopropyl butyrate, limonene, 4-penten-1-ol, cis-2-pentenol, 1-hexanol, nerol, 3-hexen-1-ol, and *E, E*-2,6-nonadienal were rapidly produced by the ‘*Tainong*’ mango in storage after 7 d, while alpha-copaene, 2-methyl-4-heptanone, 1-butanol, alpha-terpineol, linalool, and acetaldehyde contents decreased. In addition, 2-carene, cis-2-pentenol, cis-2-hexen-1-ol, 1-octanol, beta-caryophyllene, beta-pinene, alpha-phellandrene, beta-cadinene, 4-hydroxypentan-2-one, propyl butyrate, and 3-methyl-2-butenylacetat were rapidly produced in the ‘*Hongyu*’ mango after 11 d, while cis-3-hexenal, hexanal, *(E)*-2-butenal, 2-butenal, 3-hexen-1-ol, nerol, (3S)-3-methylpentan-1-ol, 1-heptanol, cyclobutanol, limonene, 2-methylprop-2-enylbenzene, β-phellandrene, and beta-elemene decreased after nine days of storage.

### 2.5. Characteristic Volatile Components Analysis

Based on the results of GC-MS and GC-IMS, a supervised OPLS-DA model was developed and the goodness-of-fit (R^2^) and predictive goodness (Q^2^) of both models exceeded 0.97, indicating that both models were accurate and robust. The variables with VIP values were also considered as characteristic VOCs to assess the identification of each volatile component for mangoes at different storage periods [32,34,35].

**Figure 5:**
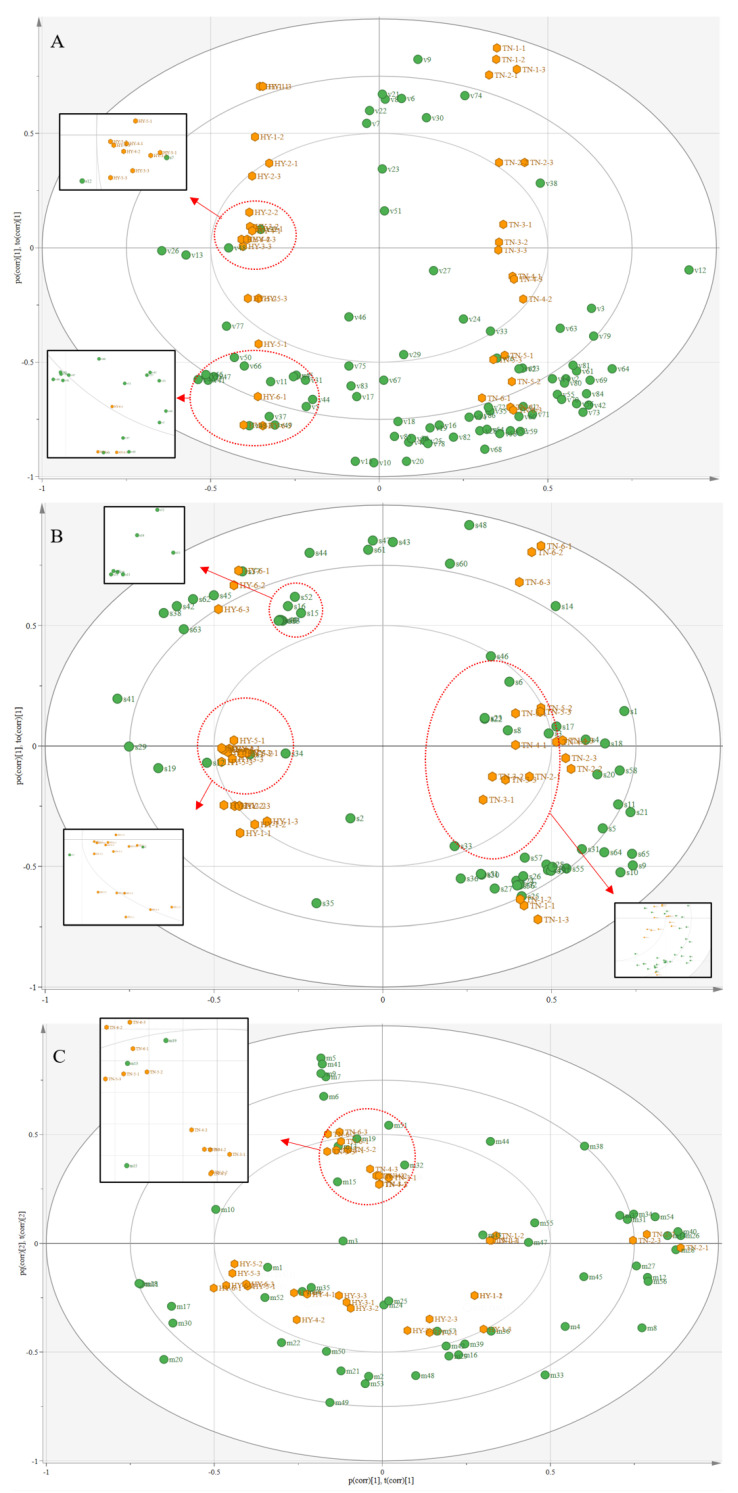
The OPLS-DA model is based on the VOCs of mangoes at different storage periods. (**A**) Biplot based on GC-IMS intact mango data, numbers “v1”–”v86” are VOCs in Table 2; (**B**) Biplot based on GC-IMS cut mango data, numbers “s1 “–“s64” for VOCs in Table 2; (**C**) Biplot of HS-SPME-GC-MS data, numbers “m1”–“m56” are the VOCs in Table 2.

Figure 5A and Table 2 show 17 characteristic VOCs identified on the basis of GC-IMS intact mango. Among them, seven characteristic VOCs of the ‘*Hongyu*’ mango—propyl propanoate, propyl butyrate, ethyl 3-methylbutanoate, benzyl alcohol, ethyl acetate, 3-octanol, and ethanol—were related to the fruity aroma of mango [36]. In contrast, ten characteristic VOCs of the ‘*Tainong*’ mango—terpinolene, pentanoic acid, myrcene, pentanoic acid, 1-octene, 3-methylbutanal, 3-pentanone, 2-hexenal, beta-ocimene, and 2-pentenal—were related to the terpene, lemon, and green aromas of mango [5,29]. Similarly, Figure 5B and Table 2 show that 18 characteristic VOCs identified on the basis of GC-IMS cut mango, of which three characteristic VOCs of the ‘*Hongyu*’ mango—propyl butyrate, 3-hydroxy-2-butanone, and methyl hexanoate—were related to the apple and fruity aromas of mango [29]. Fifteen characteristic VOCs of the ‘*Tainong*’ mango—beta-ocimene, terpinolene, limonene, 1-pentanol, 3-methylbutanol, alpha-terpinene, *(Z)*-3-hexenyl acetate, *(E)*-2-Hexenal, iso-propanol, ethyl hexanoate, 1-Hexanol—were related to the pine resinous, terpene, lemon, fatty-grassy, green-fruity notes, and green aromas of mango [5,14,29].

Correspondingly, as can be seen in Table 2, 26 characteristic VOCs were identified on the basis of GC-IMS cut mango. Figure 5C displays 12 characteristic VOCs of the ‘*Hongyu*’ mango—propyl butyrate, 4-ethyl 2-methylbutyrate, butyl hexanoate, eudesm-4-en-11-ol, alpha-terpinene, 3-hexen-1-ol, 3-hexenal, 3-carene, and 1-hexanol—were related to the sweet and fruity aromas of mango [37]. Fourteen characteristic VOCs of the ‘*Tainong*’ mango—1-methyl-3-propan-2-ylbenzene, benzaldehyde, beta-ocimene, linalyl acetate, 2-carene, 3-methylcyclohex-3-en-1-one, beta-caryophyllene, alpha-phellandrene, beta-pinene, alpha-pinene, limonene, hexanal, delta-carene, and 2-methyl Furan—were related to the grassy, floral with neroli oil, strong pine-like, turpentine, terpene and lemon aromas of mango [5,14,29,36].

Notably, propyl butyrate was identified as a characteristic VOC of the ‘*Hongyu*’ mango in both HS-SPME-GC- MS and GC-IMS models. As seen in Table 1, propyl butyrate had fruity, banana and pineapple aromas of mango, and propyl butyrate accumulated during later storage with the highest levels at 11 days. Limonene and beta-ocimene were identified as characteristic VOCs of the ‘*Tainong*’ mango in both HS-SPME-GC- MS and GC-IMS models. Among that, beta-ocimene had highly grassy aromatics, the main VOCs of mangoes [14,28,33]. This explains the strong grassy aroma of the ‘*Tainong*’ mango, and the strong fruity and sweet aromas of the ‘*Hongyu*’ mango.

## 3. Materials and Methods

### 3.1. Materials

The mango cultivars ‘*Hongyu*’ and ‘*Tainong*’ were obtained from a mango plantation (Changjiang, Hainan province, China); the planted area was approximately 53.36 km^2^. The selected more than 600 mangoes from the orchard, with each fresh weight of mango post-harvest equaling 215.9 ± 4.1 g (*n* = 30). The harvested fruits were transported to the post-harvest laboratory of Hainan University within 6 h at a temperature of 25 °C and relative humidity of 50–60%. Mango fruits of uniform shape and size and without blemishes were selected for the experiments. Six mangoes were placed in each box with air holes for ripening at room temperature (23 °C). During storage, mangoes were randomly selected for sampling every two days until they became overripe (11 d). The samples were immediately quenched with liquid nitrogen and stored at −80 °C for further analysis. The samples were labelled from 1–11 d.

### 3.2. Firmness Determination

Firmness was determined by measuring the required force when the probe was first pressed down after touching the sample using a Texture Analyser and a 2 mm diameter cylindrical planar probe (Stable Micro Systems Texture Analyser, model TA-XT plus, Surrey GU7 1YL, UK). The compression depth was 5 mm, the cylindrical planar speed of the probe was approximately 3 mm s^−1^, and the probe was held perpendicular to measure the firmness in four different locations throughout the equatorial zone of each mango.

### 3.3. Colour Measurement

To determine the colour of each mango peel, two locations on either side of the equatorial region were selected using a Chroma meter CR-400 (EC Minolta, Japan), and the values were expressed as L*, a*, and b* values based on the CIE system. L* indicates lightness (positive numbers tend to be white and negative numbers tend to be black), a* indicates the red-green value of chromaticity (positive numbers are red and negative numbers are green), and positive and negative values of b* indicate yellow and blue, respectively [1].

### 3.4. Respiration Rate and Ethylene Production

The measurement of CO_2_ and ethylene allows the calculation of respiration rate and ethylene production [1]. We chose six mango per box (boxes were perforated with air holes and each box contained six mangoes), then six mangoes were randomly selected and sealed inside an airtight pot of known volume (2.250 L). The samples were then incubated at 23 ± 1 °C for 30 min. Gas samples from the headspace of each pot were injected into an infrared gas analyser to detect the release of CO_2_ in a static system. The respiration rate was then calculated using the container volume, CO_2_ concentration difference, and fruit weight [9]. The respiration rate was expressed as nmol kg^−1^ s^−1^.

Similarly, samples in sealed airtight pots were incubated at 23 ± 1 °C for 1 h. Then 5 mL of the gas samples were collected from each airtight pot into a headspace bottle. Ethylene concentration was measured using a 2 mL syringe, and 1 mL of gas sample from the headspace of the bottle was injected into a GC (equipped with HP-5 MS and FID) system. The injection, column, and detector temperatures were set to 600 °C, 120 °C, and 250 °C, respectively. The N_2_ (carrier gas), H_2_, and airflow rates were set as 0.023 mL s^−1^, 0.67 mL s^−1^, and 6.67 mL s^−1^, respectively. The ethylene release was expressed as nmol kg^−1^ s^−1^ [38].

### 3.5. Measurement on the Soluble Solid Concentration (SSC) and Titratable Acidity

Three groups of nine fruits from each replicate were created at random, with samples from each group being mixed and squeezed through a cheesecloth to extract the juice. The concentration of soluble solids was determined using a refractometer (Master-M; Atago, Japan), then the extracted juice was placed on the refractometer’s glass prism and readings were recorded to obtain the total soluble solids (%).

Titratable acidity was used to determine the titration method. We placed 10 g of well-mixed samples into a 0.1 L volumetric bottle, added distilled water up to the scale mark, and agitated the bottle. The solution was filtered after standing for 30 min. We added two drops of phenolphthalein (1%) to 20 mL of the filtrate as an indicator and titrated it against standardised NaOH until the solution changed to pink, which did not fade within 30 s (pH = 8.1~8.3); the amount of NaOH was recorded (repeated thrice). Distilled water was used as a blank control instead of the titration sample. The titratable acidity (%) was expressed as a percentage of citric acid [39].

### 3.6. GC-IMS Analysis

To investigate the VOC composition in the ripening mango fruit, we tested the samples of intact fruit (complete fruit with peels) and cut fruit (only pulps without peels) to determine the differences between them. The intact mangoes were immediately placed in a gas sample bag to be tested for VOCs, and the cut mangoes were cut into small pieces for VOC testing. The cut mango samples (5 g) were placed in a 20 mL headspace bottle using an automatic headspace injection system. The automated headspace sampler conditions were as follows: incubation temperature, 40 °C; incubation time, 20 min; headspace injection volume, 500 μL; injection needle temperature, 85 °C; and shaking heating.

For analysis, GC-IMS equipment (FlavourSpec, G.A.S. mbH, Dortmund, Germany) was used to isolate and analyse VOCs. Chromatographic separation was performed on a FS-SE-54-CB-1 (15 m × 0.53 mm, 0.5 μm) capillary column kept at 60 °C, and the carrier gas was ultrapure nitrogen (purity ≥ 99.999%).

For cut fruit detection, the carrier gas flow rate was set at 2 mL min^−1^ for 2 min, and further increased after 2 min, increased to 10 mL min^−1^ at 10 min, and further increased to 100 mL min^−1^ at 20 min. Finally, it was elevated to 150 mL min^−1^ at 30 min; the total detection time was 30 min. For intact fruit detection, the carrier gas flow rate was set at 5.0 mL min^−1^ for 2 min, increased to 10 mL min^−1^ at 10 min, and further increased to 90.0 mL min^−1^ at 20 min; finally, it was elevated to 90 mL min^−1^ at 30 min; the total detection time was 30 min.

### 3.7. HS-SPME-GC-MS Analysis

Samples were extracted according to a previously described method [4]. Nonyl acetate was used as the pure standard compound and the peak area of the VOCs in the quantitative data was used. In brief, samples (mango pulp, 5 g) were placed in a 20 mL headspace bottle, then a manually operated headspace sampling apparatus was used with a 50/30 μm DVB/CAR/PDMS fibre (PerkinElmer, Waltham, MA, USA). After the sample was pre-equilibrated at 40 °C for 10 min, extraction in headspace vials was performed for 30 min at the same temperature. Once the VOCs were extracted, they were placed in the GC-MS injection port for thermal desorption, which took place at 230 °C for 1 min.

We used a GC-MS instrument (Clarus 690-SQ 8 T) with a gas chromatographic column HP-5MS (30 m × 0.25 mm × 0.25 μm, Agilent, Santa Clara, CA, USA). The injection was conducted in mode at 250 °C. Helium was employed as the carrier gas with an average linear flow rate of 1.0 mL min^−1^. Chromatographic separations were performed as follows: 40 °C for 0–6 min; increased to 160 °C at a rate of 3 °C min^−1^ and held for 2 min; then increased to 230 °C at a rate of 10 °C min^−1^ and held for 5 min. The scan mode at 30–450, EI at 70 eV, interface temperature of 200 °C, and ion source temperature of 220 °C were used for mass-selective detection.

### 3.8. Statistical Analysis

The HS-GC-IMS and HS-SPME-GC-MS data were standardised and one-way ANOVA was performed using SPSS23 (SPSS Inc., Chicago, IL, USA). The software’s integrated NIST and IMS databases were utilised for qualitative substance analysis using the Reporter and Gallery plug-in programs to build the difference and fingerprint of the substances analysed. Origin 2021 software was used for the drawing and Simca 14.1 software was used for OPLS-DA.

## 4. Conclusions

In this study, GC-IMS and GC-MS analyses were used to identify VOCs in ‘*Hongyu*’ and ‘*Tainong*’ mangoes during storage. GC-IMS identified 43 VOCs in cut mangoes and 52 VOCs in intact mangoes. A total of 94 VOCs were detected using GC-MS. The identified substances were mainly esters and terpenes, with a few aldehydes and alcohols. The results of the OPLS-DA analysis showed that the characteristic VOCs of terpene levels in the ‘*Tainong*’ were higher than those in the ‘*Hongyu*’, and the ester levels in the ‘*Hongyu*’ were higher than those in the ‘*Tainong*’. In addition, the results of OPLS-DA clearly showed that beta-ocimene was the characteristic VOC that distinguished the ‘*Tainong*’ mango, and propyl butyrate was the characteristic VOC that distinguished the ‘*Hongyu*’ mango, by both GC-IMS and GC-MS. The contents of VOCs in cut fruit mangoes showed intermittent changes, and the contents of most VOCs in intact mangoes showed a gradual increase over time. The VOCs content in intact mangoes was higher than in the cut fruits. This is consistent with the actual sensory aroma of the two mango products. Analysis of VOCs for mangoes in storage will benefit the mango industry by improving their storage management, achieving better quality, and reducing food waste. Mangoes from days five to seven were considered commercially mature. Both GC-MS and GC-IMS showed that propyl butyrate accumulated during later storage and was considered a symbol of poor fruit quality. This observation will be useful for understanding the ripening processes of mangoes and provides guidelines regarding the appropriate time to market them.

## Figures and Tables

**Table 1:** The marker VOCs of ‘*Tainong*’ and ‘*Hongyu*’ mango and their potential contributions to mango aroma.

Name	Sample with the Highest Content (%)					
	Intact		Cut			
	HS-GC-IMS	HS-GC-IMS	HS-GC-IMS	HS-GC-IMS	HS-GC-MS	HS-GC-MS
	*Hongyu*	*Tainong*	*Hongyu*	*Tainong*	*Hongyu*	*Tainong*
Trimethylpyrazine	11 d (0.04)	11 d (0.15)				
Terpinolene	11 d (0.56)	11 d (0.85)	1 d (0.89)	1 d (1.78)	1 d (5.60)	1 d (7.08)
Sabinene					5 d (0.03)	9 d (0.14)
Propyl propanoate			11 d (0.84)	11 d (0.4)		
Propyl butyrate	11 d (0.21)	11 d (0.04)	11 d (0.04)	11 d (0.10)	11 d (0.01)	11 d (0.02)
Propyl acetate	5 d (0.07)	5 d (0.04)				
Propan-2-one	3 d/5 d (0.09)	11 d (0.17)				
Phenethyl butyrate					1 d (0.01)	-
Pentyl acetate			-		7 d/11 d (0.05)	11 d (0.05)
Pentanoic acid			5 d (0.07)	1 d (0.08)		
Nerol					9 d (0.04)	9 d (0.05)
Myrcene			1 d (0.10)	1 d (0.50)	3 d (0.30)	3 d (0.40)
Methyl hexanoate	11 d (0.04)	11 d (0.01)				
Methyl butanoate	11 d (0.52)	11 d (0.36)	11 d (0.01)	-	-	11 d (0.02)
Methyl acetate					3 d (0.02)	-
Methyl crotonate			11 d (0.15)	11 d (0.03)	-	-
Linalyl formate					-	5 d (0.03)
Linalyl acetate						9 d (0.13)
Linalool						5 d (0.05)
Limonene	11 d (0.11)	11 d (0.18)	1 d (0.07)	1 d (0.47)	3 d (0.64)	3 d (0.91)
Isopropyl butanoate			11 d (0.65)	11 d (0.72)	-	7 d (0.09)
Isopentyl alcohol			9 d (0.02)	7 d/9 d (0.02)		
Isobutanal	11 d (0.01)	11d (0.05)				
Isoamyl acetate	3 d/5 d (0.03)	3 d/5 d (0.07)				
Humulene					3 d (0.10)	3 d (0.08)
Hexyl acetate	9 d/11 d (0.02)	3 d/5 d/7 d/9 d/11 d (0.02)				
Hexene					7 d (0.02)	-
Hexanal	5 d (0.58)	11 d (0.54)			1 d/3 d/9 d (0.02)	1 d (0.03)
Heptanal	9 d/11 d (0.01)				9d (0.01)	-
Ethyl pyruvate	3 d/5 d/7 d/9 d/11 d (0.02)	3 d/5 d/7 d/9 d/11 d (0.02)				
Ethyl propanoate	11 d (0.39)	11 d (0.44)	11 d (0.69)	11 d (0.14)		
Ethyl pentanoate	11 d (0.08)	11 d (0.02)				
Ethyl octanoate	9 d (1.56)	7 d (2.29)				
Ethyl isobutyrate	11 d (0.12)	5 d (0.04)				
Ethyl hexanoate	11 d (0.92)	7 d (0.99)				
Ethyl heptanoate	7 d/9 d/11 d (0.02)	7 d (0.03)				
Ethyl butyrate	11 d (1.41)	11 d (1.47)	11 d (0.45	11 d (0.68)		
Ethyl acetate	11 d (1.03)	11 d (0.91)	11 d (1.84)	11 d (0.82)	1 d (0.02)	11 d (0.01)
Ethyl 3-methylbutanoate	11 d (0.27)	3 d (0.25)	11 d (0.03)	-	-	-
Ethyl 2-methylpropanoate			11 d (0.01)	-		
Ethyl 2-methylbutanoate	11 d (0.18)	7 d (0.19)	11 d (0.01)	-	3 d/7 d (0.01)	-
Ethanol	1 d (1.56)	1 d (1.36)	9 d (5.47)	11 d (5.42)		
Ethanal	3d/5d/11d (0.04)	9d/11d (0.08)				
Delta-carene			1 d (0.70)	1 d (0.66)	1 d (0.15)	3 d (0.09)
Decanal					9 d (0.03)	1 d (0.02)
Cyclohexyl formate					11 d (0.04)	-
Citronellol			5 d (0.04)	-		
Carveol					3 d (0.01)	1 d (0.02)
Butyl propanoate	9d/11d (0.04)	11d (0.08)				
Butyl hexanoate					-	11d (0.05)
Butyl butanoate	11d (0.39)	5d/9d (0.05)				
Butyl acetate	5d (0.21)	5d (0.19)				
Butanal	1d (0.03)	1d (0.02)				
Butan-2-one	1d (0.01)	1d (0.01)				
Beta-selinene					1d (1.48)	1d (0.02)
Beta-pinene	9d (0.35)	11d (0.36)	1d (0.07)	1d (0.06)	1d (0.16)	3d (0.33)
Beta-ocimene	11d (0.06)	11d (0.17)	1d (0.77)	1d (0.73)	5d (0.51)	1d (0.41)
Beta-caryophyllene					11 d (0.49)	3 d (0.19)
Benzyl alcohol			5 d (0.04)	-		
Alpha-terpinene	11 d (0.33)	11 d (0.63)	1 d (0.05)	1 d (0.41)	5 d (1.65)	3 d (1.23)
Alpha-pinene	11 d (0.10)	11 d (0.12)	1 d (0.14)	1 d (0.43)	7 d (1.41)	3 d (1.12)
Alpha-phellandrene	11 d (0.17)	9 d (0.26)	1 d (0.10)	1 d (0.45)	11 d (0.03)	1 d (0.08)
Alpha-ocimene					1 d (0.02)	-
Alpha-cubebene					7 d (0.04)	5 d/9 d (0.04)
Alpha-copaene					1 d (0.07)	1 d (0.20)
Alloaromadendrene					11(0.04)	3 d (0.02)
Acetone					1 d/3 d (0.01)	
4-Penten-1-ol						11 d (0.05)
4-Methyl-2-pentanone			-	9 d (0.02)		
3-Pentanone			3 d (0.13)	-		
3-Octanol			5 d/7 d/9 d (0.01)	-		
3-Methylbutanol	11 d (0.12)	5 d (0.21)				
3-Methylbutanal			-	9 d (0.12)		
3-Methyl-4-heptanone						
3-Methyl-2-butanol	11 d (0.07)	11 d (0.07)				
3-Methyl-1-pentanol			11 d (0.06)	11 d (0.01)		
3-Hydroxy-2-butanone			7 d/9 d (0.02)	11 d (0.02)		
3-Hydroxy-2-butanone	11 d (0.17)	11 d (0.15)				
3-Hexenal					9 d (0.1)	7 d (0.08)
3-Hexen-1-ol					7 d/9 d (0.03)	11 d (0.02)
3-Hexanone					9 d (0.49)	
3-Carene					1 d (2.41)	3 d (1.55)
2-Pentanone		11 d (0.03)	-	11 d (0.06)		
2-Methylpent-4-enal					7d/9d (0.06)	7d (0.04)
2-Methyl-1-propanol	11 d (0.07)	5 d (0.07)				
2-Methyl butanol			11 d (0.01)	-		
2-Ethyl-1-hexanol			5 d (0.03)	-		
2-Carene					1 d (2.58)	1 d (5.88)
2-Acetylthiazole	7 d (0.02)					
2,3-Pentanedione	11 d (0.05)	11 d (0.12)				
1-Propanol	1 d (0.09)	1 d (0.04)				
1-Octene			1 d (0.07)	3 d (0.19)		
1-Methylethyl acetate	5 d/7 d/9 d/11 d (0.08)	11 d (0.11)				
1-Methyl-3-propan-2-ylbenzene					1 d (0.10)	1 d (0.11)
1-Hydroxy-2-propanone			-	9 d (0.05)		
1-Hexanol	11 d (0.03)	9 d (0.03)				
1-Butanol	5 d/7 d/11 d (0.03)	3 d (0.03)				
*(Z)*-rose oxide			5 d (0.03)	-		
*(Z)*-hex-3-en-1-ol					11 d (0.07)	
*(Z)*-3-hexenyl acetate	11 d (0.05)	11 d (0.08)				
*(Z)*-2-hexen-1-ol					11 d (0.03)	
*(E, Z)*-2,6-nonadienal			-	9 d (0.15)	11 d (0.03)	11 d (0.02)
*(E)*-beta-ocimene			-	1 d (0.05)		
*(E)*-2-pentenal			-	9 d (0.04)	11 d (0.03)	11 d (0.03)
*(E)*-2-nonenal			5 d (0.12)	1 d (0.07)		
*(E)*-2-hexenal		11 d (0.11)	-	9 d (0.10)	11 d (0.08)	11 d (0.03)
*(2E,6Z)*-nona-2,6-dienal					11 d (0.03)	-

**Table 2:** Calculating the variable importance of projection (VIP > 1).

GC-IMS Intact Mango			GC-IMS Cut Mango			GC-MS		
No.	Compounds	VIP	No.	Compounds	VIP	No.	Compounds	VIP
s65	Terpinolene	2.08753	v64	3-Methylbutanol	1.88659	m20	Propyl butyrate	1.64887
s58	Pentanoic acid	1.91624	v69	Beta-ocimene	1.76667	m49	Alpha-terpinene	1.5228
s56	Myrcene	1.5574	v79	1-Pentanol	1.75097	m53	eudesm-4-en-11-ol	1.34255
s57	Pentanoic acid	1.46329	v84	Trimethylpyrazine	1.74168	m48	4-Methylvalerophenone	1.32299
s10	1-Octene	1.27127	v73	Terpinolene	1.72661	m30	Hexene	1.2544
s18	3-methylbutanal	1.15562	v56	Alpha-terpinene	1.69203	m21	4-Ethyl 2-methylbutyrate	1.24467
s21	3-Pentanone	1.11363	v61	*(E)*-2-Hexenal	1.66245	m18	1-Hexanol	1.21866
s1	*(E)*-2-hexenal	1.10303	v81	*(Z)*-3-hexenyl acetate	1.61886	m2	Acetaldehyde	1.21332
s5	*(E)*-beta-ocimene	1.03414	v12	Iso-propanol	1.46001	m33	3-Carene	1.15313
s4	*(E)*-2-Pentenal	1.01497	v60	Limonene	1.39653	m17	3-Hexen-1-ol	1.13545
s62	Propyl propanoate	1.75477	v68	Ethyl hexanoate	1.22739	m11	3-Hexenal	1.02138
s61	Propyl butyrate	1.26807	v72	Hexyl acetate	1.2223	m9	Butyl hexanoate	1.29179
s41	Ethyl 3-methylbutanoate	1.18943	v86	Ethyl octanoate	1.21327	m38	1-methyl-3-propan-2-ylbenzene	1.40181
s29	Benzyl alcohol	1.14882	v76	3-Methyl-3-buten-1-ol	1.18841	m5	Benzaldehyde	1.41035
s42	ethyl acetate	1.0974	v82	1-Hexanol	1.10373	m41	beta-ocimene	1.31592
s19	3-Octanol	1.03367	v65	Propyl butyrate	1.52596	m7	Linalyl acetate	1.21866
s38	ethanol	1.01365	v77	3-Hydroxy-2-butanone	1.43828	m44	2-Carene	1.21027
			v57	Methyl hexanoate	1.15345	m26	3-methylcyclohex-3-en-1-one	1.19349
						m54	Beta-caryophyllene	1.15453
						m32	Alpha-phellandrene	1.13682
						m28	Beta-pinene	1.11746
						m34	Alpha-Pinene	1.05874
						m40	Limonene	1.04677
						m12	Hexanal	1.01841
						m31	Delta-carene	1.01799
						m6	2-Methyl Furan	1.00994

## Data Availability

Not applicable.

## Sample Availability

Samples of the compounds are available from the authors.
